# Development and evaluation of training programs to improve health checkup recommendation materials

**DOI:** 10.3389/fpubh.2025.1540529

**Published:** 2025-09-03

**Authors:** Runa Ogawa, Hirono Ishikawa, Yoshiharu Fukuda

**Affiliations:** Teikyo University Graduate School of Public Health, Tokyo, Japan

**Keywords:** health checkups, insurer, health insurance, behavior change, public health

## Abstract

**Background:**

Health communication materials must be easily understood by the target readers. Although numerous efforts have been made to recommend preventive services, the training of practitioners to create effective recommendation materials is insufficient. This study verifies whether the training provided to practitioners could lead to improvements in the recommendation materials using a checklist based on the suitability assessment of materials (SAM).

**Methods:**

This study targeted the public health insurers in Tokyo. Individual and group training was provided to improve the recommendation materials of specific health checkups using a checklist with reference to the SAM. The materials (flyers, postcards, leaflets, etc.) created by the insurers were evaluated by six randomly assigned evaluators. A suitability score indicating the appropriateness of the material was calculated using an evaluation manual to verify the improvements in the materials before and after the training.

**Results:**

Of the 49 insurers who participated in the training, 31 evaluated the materials both before and after the training. The mean suitability score [standard deviation] increased from 48.6 [7.9] before training to 51.6 [8.7] after training, although the difference was not statistically significant (*p* = 0.09). However, statistically significant increases were observed in four items: information essential for undergoing health checkups, clear titles and captions that explain graphic content, consistent and readable layout, and sufficient margins and line spacing.

**Conclusion:**

SAM-based training led to limited improvements in the recommendation materials created by insurers. Providing more thoughtfully designed training to insurers is expected to increase effective health communication materials that encourage recipients to take action.

## 1 Introduction

### 1.1 Health communication materials

Supporting behavioral change in individuals requires that the materials used for behavioral interventions are easy to read and understand. People with low health literacy are known to inappropriately use healthcare services, poorly manage chronic conditions, and use medical services inefficiently (1–3). Therefore, clarity and readability are essential in the guidance materials used to promote participation such as in health checkups; however, increasing the participation rate remains a key challenge. Previous studies have shown that improving the readability of screening invitations can increase the participation rates (4). Furthermore, creating easy-to-read health information not only makes it easier for the reader to understand but also enhances self-efficacy in performing health behaviors (5). It has also been reported that clear materials can increase the readers’ comprehension, sense of security, and satisfaction, and satisfaction positively influences decision-making (6). Of course, it is difficult to associate changes in participation rates solely with improved materials, as people’s perceptions and beliefs about health are not always rational and various factors influence their behavior. Still, improving communication materials remains a key element of public health interventions.

Practical guidelines for creating easy-to-understand health and medical materials in English (7) and practical kits to support the creators (8) are available. While similar support for creating health and medical materials in Japanese is not well-established, principles for crafting clear and persuasive health information have been established based on academic knowledge (9, 10) and are utilized as practical guides for creating materials in Japanese.

### 1.2 Japan’s universal coverage and national health insurance

This study focused on materials that encouraged participation in the specific health checkups and health guidance provided under the municipal National Health Insurance (NHI) system. These programs aim to promote the prevention of lifestyle-related diseases and serve as an important strategy for addressing the increasing burden of healthcare costs associated with Japan’s rapidly aging population.

In Japan, all citizens are enrolled in one of several public health insurance schemes. NHI is one such scheme, operated by municipalities and primarily covering self-employed individuals and those working in agriculture, forestry, and fisheries. Recently, insurers have been increasingly expected to improve participation rates in specific health checkups and health guidance as part of government-led efforts to control rising healthcare expenditures.

One of the most critical initiatives in disease prevention is Japan’s specific health checkup and health guidance program, mandated for public health insurers since 2008 (11). In this program, insurers aim to prevent lifestyle-related diseases such as diabetes and hypertension by identifying individuals aged 40–74 with metabolic syndrome and providing tailored counseling based on their disease risks. The national targets for participation rates have been set at 70% for specific health checkups and 45% for specific health guidance. However, most insurers have not reached these targets (12). Moreover, public subsidies are allocated according to the participation rates, providing a strong incentive for insurers to raise them. The materials evaluated in this study—printed materials such as postcards and leaflets—are a key component of those efforts, intended to inform recipients about the importance of preventing lifestyle-related diseases and to encourage participation in the program.

In this study, the term “insurer” refers to entities responsible for implementing the specific health checkups under Japan’s National Health Insurance system. These include municipal governments (cities, wards, towns, and villages) and National Health Insurance Associations operated by specific occupational groups. They are primarily public administrative bodies and do not refer to private insurance companies.

Despite the widespread distribution of recommendation materials for specific halth checkups, several issues have been identified, such as failing to attract recipients’ attention or prompt action. Improvements have been suggested regarding content, information volume, layout, timing of distribution, and audience segmentation (13). However, there remains limited evidence on effective training approaches to enhance the quality of such materials.

### 1.3 Objective of this study

To address this gap, the current study developed a checklist based on the Suitability Assessment of Materials (SAM) framework, which is used to evaluate the suitability of health communication materials (14). The SAM provides scores that categorize materials as Superior (70–100), Adequate (40–69), or Not Suitable (0–39). By adapting this tool to the perspective of material creators, we aimed to offer practical training to support the development of materials that effectively encourage participation in specific health checkups and health guidance. We evaluated changes in the clarity and readability of the materials before and after providing the training to municipal staff.

## 2 Methods

### 2.1 Development of the checklist

The checklist items were based on the Japanese version of the SAM. As presented in Table 1, the checklist consisted of 20 items. Each item was evaluated using the following criteria: excellent, good, needs some improvement, needs much improvement, and not applicable.

**Table 1:** Checklist based on Japanese version of the SAM.

Main category	Subcategory	Evaluation items
1. Content	A) Is the purpose clear in the title or others? B) Does it describe the actions/activities to solve the problem? C) Is there unnecessary information or too much information? D) Does it contain the information that the reader wants to know?	a) Is the title prominent? b) Is the purpose specific in the title and others? c) Is there information necessary for the action for health checkups? d) Is there too much information on medical facts? e) Are the benefits of undergoing health checkups explained? f) Is the cost of health checkups written? g) Is the deadline of health checkups written?
2. Literacy demand	A) Is the text easy to read? B) Is it written in a conversational style and active voice? C) Is the language and terminology too difficult?	a) Is the text easy to read? b) Is it written in a conversational style? c) Is it written in active voice? d) Is the language and terminology too difficult?
3. Graphic illustrations, lists, tables, charts	A) Is it familiar, interesting, and clearly expresses its purpose? B) Type: Is it concise and familiar to the reader? C) Relevance of content: Does it visually represent only the key points? D) Is there a title and a caption to indicate the content?	a) Is the meaning clear with favorable emphasis? b) Is it not too technical? c) Does it visually represent only the key points? d) Is there a title and a caption to indicate the content?
4. Layout and typography	A) Is the layout suitable? B) Are the type sizes and fonts suitable? C) Is the information divided into small sections with headings?	a) Is the layout consistent and readable in sequence? b) Are there sufficient margins and line spacing? c) Is the emphasis of the type not excessive? d) Is the type not too small? e) Is the information divided into small sections with headings?
5. Learning stimulation and motivation	A) Is the reader expected to think and answer questions, not one-sidedly? B) Do the readers feel able to read and understand the information, or take the desired action or activity? C) Do the expressions convey an attitude of respect for the reader as a person?	a) Is the information not one-sided? b) Do the readers feel able to receive the health checkups? c) Do the expressions convey an attitude of respect for the reader as a person?
6. Custom contextual appropriateness	A) Is the color use suitable? B) Is the contact information clear? C) Is the paper size suitable?	a) Is the color use suitable? b) Is the contact information clear? c) Is the paper size suitable?

### 2.2 Participants

The participants were member insurers of the Tokyo Metropolitan National Health Insurance Organization (Tokyo NHIO) and were responsible for developing recommendation materials for specific health checkups. The Tokyo NHIO is a legally recognized organization established with the approval of the Governor of Tokyo under Article 83 of the National Health Insurance Act for insurers (wards, cities, towns, and National Health Insurance associations). Tokyo metropolitan government, special wards (“Ku”), cities, towns, villages, and National Health Insurance Associations for specific occupational groups in Tokyo are members (84 insurers in total) of Tokyo NHIO (15).

These member insurers were represented by municipal staff who were the actual senders of health checkup recommendation materials. Specifically, they were engaged in insurance-related services. The majority were administrative officers (74.5%), followed by public health nurses (21.6%) and others, such as registered dietitians (3.9%). While 69% had practical experience in preparing notification materials, only 26% had ever received any related training. These staff members participated in the training voluntarily after a call for participation was issued to all insurers in the Tokyo area. The intended receivers of the materials were primarily individuals aged 40 to 74 who are self-employed, unemployed, or employed in part-time or non-regular positions that are not covered by employee health insurance.

### 2.3 Training

The training was conducted through individual and group sessions. In the individual sessions, a checklist was used to provide personalized feedback and specific suggestions for the improvement of the materials. The insurers were asked to submit specific health checkups and guidance recommendation materials (leaflets and postcards). Multiple researchers that are proficient in health communication reviewed these materials using the checklist developed in this study. The review included symbolic evaluations and detailed comments in the free description section.

The group sessions were organized to provide participants with hands-on experience in using the checklist. The session began with a lecture on the efforts to improve health checkup participation rates, application of behavioral science theories, and process evaluation of specific health checkups (Lecture 1). This was followed by a lecture on segmenting the target audience, clarifying the message concepts, conducting preliminary surveys, selecting communication channels, and creating messages and materials (Lecture 2). Each lecture, delivered by two experts, lasted 15 min. Subsequently, Lecture 3 covered the explanation and application of the checklist developed in this study. An exemplary case presented was a material with sufficient margins and a clear layout, placing the most important information for the recipient—such as what action to take and where to apply—in a prominent position. In contrast, problematic cases included materials that used indirect or bureaucratic expressions (e.g., “has become available”) instead of more conversational ones (e.g., “you can now use”), placed insurer-centered messages (e.g., “we need to raise our screening rate”) in the most visible areas, or used unrelated illustrations without explanatory captions, potentially confusing the reader. The session concluded with a group work activity.

### 2.4 Data collection

Insurers who participated in individual sessions, group sessions, or both were asked to submit self-produced materials (flyers, postcards, leaflets, etc.) to recommend specific health checkups and health guidance in December 2020. These materials had actually been distributed to eligible residents in their respective municipalities or other insured populations covered by the submitting insurers. The materials before (2018 version) and after training (2020 version) were collected in an electronic file format. The suitability scores of the materials were calculated before and after training.

### 2.5 Materials for analysis

Of the 84 insurers, 49 (58%) participated in training. Among them, 31 were included in the analysis, after excluding those who did not submit materials (*n* = 13), those whose materials were not collected in pairs before and after the training (*n* = 2), those who did not report changes in their materials after the training (*n* = 2), and those who reported making changes without referring to the training (*n* = 1).

A total of 62 materials (31 before and 31 after training) collected from 31 target insurers were included in the analysis.

Pre-training materials were created in fiscal year 2018. However, insurers who reported not having created materials in 2018 used materials from 2017 as pre-training materials. Post-training materials were created in fiscal year 2020. For insurers that did not create materials in 2020, materials from 2019 were used as post-training materials.

For insurers who submitted multiple materials before and after the training, we thoroughly reviewed the purpose of each material (whether it was for a specific health checkup or specific health guidance, whether it was an initial notice or reminder, or whether there was segmentation) to select one pair with the same purpose. References indicating a specific year were removed from all the materials.

### 2.6 Suitability score

The evaluation items were expanded to 26 based on the original 20 checklist items while incorporating the specific content of the specific health checkup (Table 1). Each item was rated on the following scale: excellent = 3 points, good = 2 points, needs some improvement = 1 point, and needs much improvement = 0 points. The method for calculating the suitability score was the same as that used in the original version of SAM, using the following formula: total evaluation score/maximum possible score × 100. The suitability score for each material was adjusted for individual evaluator variability by calculating an adjusted score using the following formula: 50 + ((individual evaluator’s score – average score of individual evaluator) / individual evaluator’s standard deviation) × 10. The final suitability score for each material was the average score of the three evaluators.

Each material was evaluated by three evaluators. Before the evaluation, the evaluators underwent a two-hour online training session using the “Evaluation Manual for Specific Health Checkup Recommendation Materials Based on SAM” and sample materials. The evaluators were blinded to the materials used before and after the intervention. The key allocation condition was that the same evaluator would not evaluate both the pre- and post-intervention materials from the same insurer. It was noted that there is individual variability in the evaluation of Japanese texts (16), and involving multiple evaluators could help minimize the influence of individual differences (17). Therefore, it is desirable to have as many different combinations of the three evaluators as possible. Six evaluators (labeled A-F) were randomly assigned to the materials, as presented in Supplementary Table 1.

### 2.7 Inter-rater reliability examination

The Cronbach’s alpha coefficient for the scores from the six evaluators who assessed two types of materials, which were not part of this study, was 0.56 before adjustment and 0.60 after adjustment. Owing to low reliability, we further examined the inter-rater correlation (Spearman’s correlation coefficient and significance level) among the evaluators of the 31 insurers (62 materials) included in the analysis. The correlations were as follows: Evaluators A and B (*r* = 0.78, *p* < 0.0001), A and C (*r* = 0.69, *p* = 0.02), B and C (*r* = 0.80, *p* < 0.0001), and E and F (*r* = 0.53, *p* = 0.01). Evaluator D did not exhibit significant correlations with any of the other evaluators.

Therefore, in addition to analyzing the changes in the suitability scores for the materials before and after training using the results from all six evaluators, we conducted a sub-analysis excluding Evaluator D using the scores from the remaining five evaluators.

### 2.8 Statistical analysis

The means and standard deviations of the suitability scores for the “pre-training” and “post-training” materials were calculated and compared using a paired *t*-test (two-tailed). The differences in the mean values were also compared for each item in the evaluation manual. Statistical analyses were performed using the SAS software (Ver. 9.4), with a significance level of 5%.

**Figure 1:**
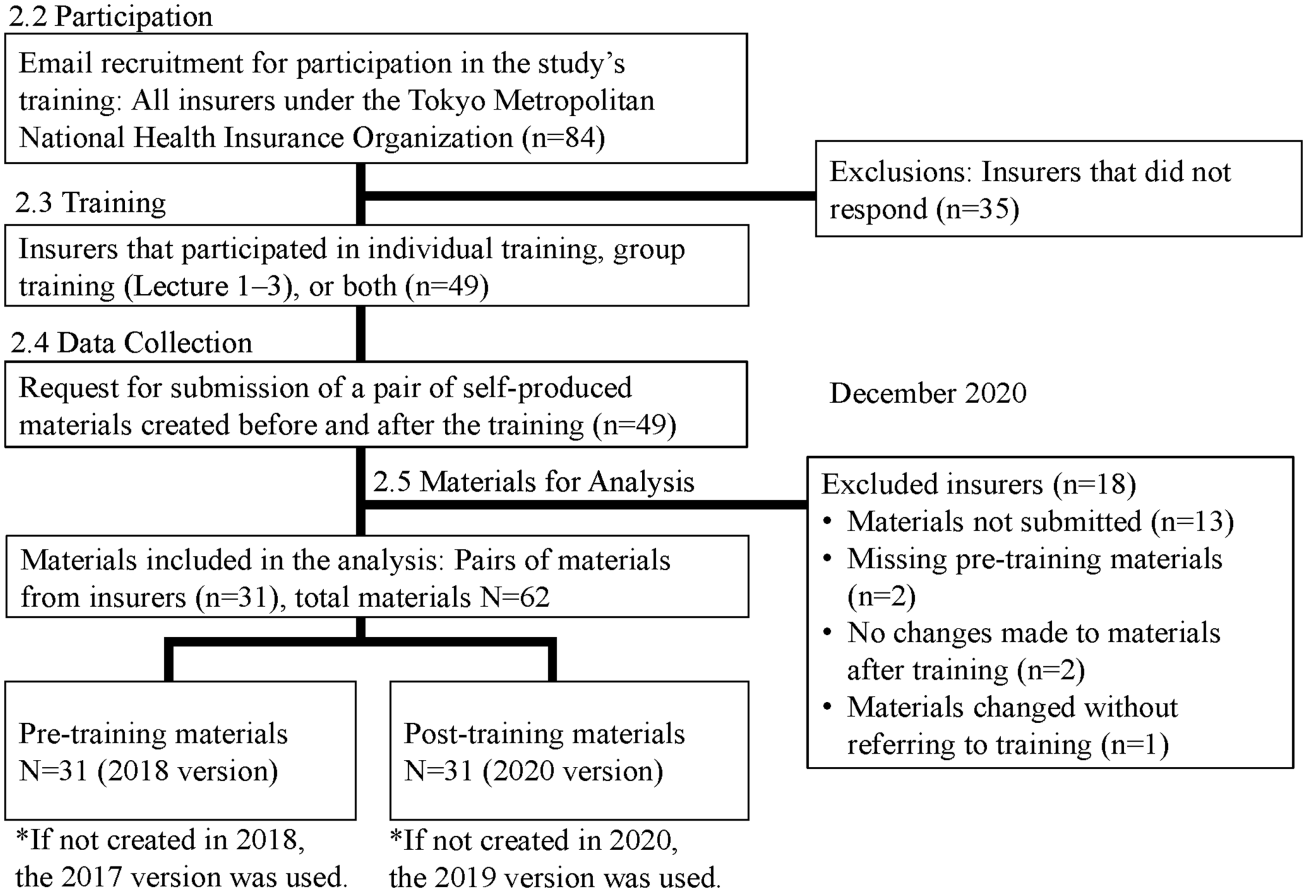
Research outline.

This study adheres to standard reporting guidelines for research involving non-human subjects. In Japan, research such as ours—evaluating the clarity of health checkup recommendation materials (e.g., flyers, postcards, leaflets)—is not subject to review under the “Ethical Guidelines for Medical and Biological Research Involving Human Subjects.” As the work did not involve human participants but rather focused on organizational practices, institutional ethics committee or IRB approval was not required. Instead, an explanatory document was distributed to health insurers to notify them of the intent to evaluate the effectiveness of revised materials (Figure 1).

## 3 Results

The mean suitability score [standard deviation] increased from 48.6 [7.9] before training to 51.6 [8.7] after training. The mean difference between pre- and post-training was 3.0 (SD 9.4), which was not statistically significant (*p* = 0.09).

In the sub-analysis excluding Evaluator D, who showed no significant correlation with any other evaluator, the mean suitability score before training was 48.3 (SD 8.1), and 52.0 (SD 9.2) after training. The mean difference between pre- and post-training was 3.7 (SD 10.0), which was statistically significant (*p* = 0.049).

Table 2 lists the suitability scores for each item before and after training. Among the 26 items, four showed statistically significant differences: “1. c) Is there information necessary for the action for health checkups?” (*p* = 0.0496), “3. d) Is there a title and caption to indicate the content of graphics?” (*p* = 0.03), “4. a) Is the layout consistent and readable?” (*p* = 0.04), and “4. b) Are there sufficient margins and line spacing?” (*p* = 0.046).

**Table 2:** Comparison of suitability scores for materials before and after training (by item).

Evaluation items	Before* 31 materials	After* 31 materials	*p*-value†
1. Content			
a) Is the title prominent?	53.8 (21.7)	56.2 (23.0)	0.61
b) Is the purpose specific in the title and others?	57.7 (22.2)	56.5 (26.3)	0.84
c) Is there information necessary for the action for health checkups?	50.7 (20.8)	59.5 (18.0)	0.0496
d) Is there too much information on medical facts?	63.0 (18.2)	61.4 (24.5)	0.73
e) Are the benefits of undergoing health checkups explained?	29.3 (24.7)	29.2 (25.4)	0.98
f) Is the cost of health checkups written?	47.2 (31.0)	44.0 (34.3)	0.69
g) Is the deadline of health checkups written?	46.6 (31.0)	41.7 (38.7)	0.40
2. Literacy demand			
a) Is the text easy to read?	36.4 (10.5)	38.2 (17.2)	0.71
b) Is it written in a conversational style?	67.7 (20.7)	72.0 (8.03)	0.06
c) Is it written in active voice?	68.4 (8.3)	72.6 (9.13)	0.12
d) Is the language and terminology too difficult?	48.2 (9.4)	50.5 (24.1)	0.66
3. Graphic illustrations, lists, tables, charts			
a) Is the meaning clear with favorable emphasis?	28.5 (21.6)	31.9 (18.5)	0.50
b) Is it not too technical?	66.5 (16.8)	64.8 (17.9)	0.67
c) Does it visually represent only the key points?	58.4 (18.9)	60.1 (16.0)	0.80
d) Is there a title and a caption to indicate the content?	27.7 (28.9)	43.8 (26.9)	0.03
4. Layout and typography			
a) Is the layout consistent and readable in sequence?	51.5 (21.5)	62.0 (15.8)	0.04
b) Are there sufficient margins and line spacing?	42.6 (25.8)	54.6 (19.7)	0.046
c) Is the emphasis of the type not excessive?	41.6 (32.6)	46.4 (32.9)	0.39
d) Is the type not too small?	41.7 (20.1)	42.1 (23.3)	0.92
e) Is the information divided into small sections with headings?	62.1 (15.3)	59.6 (16.5)	0.58
5. Learning stimulation and motivation			
a) Is the information not one-sided?	11.7 (26.8)	13.4 (30.4)	0.82
b) Do the readers feel able to receive the health checkups?	45.2 (13.9)	47.8 (20.1)	0.44
c) Do the expressions convey an attitude of respect for the reader as a person?	63.4 (12.7)	59.3 (19.5)	0.30
6. Custom contextual appropriateness			
a) Is the color use suitable?	42.6 (27.7)	53.1 (21.2)	0.11
b) Is the contact information clear?	60.4 (22.2)	65.8 (22.8)	0.12
c) Is the paper size suitable?	49.8 (20.2)	52.9 (16.5)	0.50

Bold values indicate the best performance for each metric. *Mean (standard deviation), †Paired *t*-test (two-sided).

## 4 Discussion

In the evaluation of the recommendation materials for specific health checkups and health guidance in Tokyo, the suitability scores increased after the training, although the difference was not statistically significant. However, among the individual items, statistically significant improvements were observed in four areas: items related to the information necessary for undergoing health checkups, titles and captions to indicate graphic content, consistent and readable layout, and sufficient margins and line spacing, indicating that the training led to limited improvements in the clarity of the materials.

In this study, we evaluated the basic elements indicated in U.S.-developed guidelines for writing clear materials (18). However, rewriting the materials may require additional skills. For example, even if the content is the same, changing the size of the paper or using complex color schemes could potentially make the materials more difficult to understand. Recommendation materials for specific health checkups are created annually; therefore, knowledge and skills regarding rewriting the existing materials are necessary. Additionally, skills in developing messages that consider the characteristics of the target audience (19) are essential, as is understanding how to write for different devices, such as the web or mobile devices, where display space is more limited than in print. The aforementioned guidelines, along with the U.S. government’s “Guidelines for Effective Writing” and “Writing for the Web” (20), include aspects not covered by the SAM, such as techniques for summarizing what the reader should do from the content and presenting it in an easy-to-read table format, aligning the perspectives of the reader and the writer, and training to start with the issue most important to the reader, rather than the background. These were not fully conveyed in the training provided in this study and should be incorporated into future training programs to strengthen advanced health writing techniques.

This study has several limitations. First, the variability in coder ratings should be noted. Although all six coders—three health professionals and three non-health professionals—underwent training using a detailed manual and sample materials, one coder (D) showed low correlation with the others. D is an expert in both public health and marketing, and was the only one among the six coders with professional experience in advertising in the private sector. This unique background may have influenced their evaluation tendencies. The second limitation was the potential for selection bias. The insurers who participated in this study voluntarily requested training, suggesting that they may have had a higher awareness of information provision. A comparison with insurers who did not participate in training would be desirable. Third, this study was conducted before the COVID-19 pandemic. However, the specific health checkup and health guidance have continued unchanged even in the post-pandemic period. A survey by the Tokyo Metropolitan Government in 2023 reported that 82.2% of insurers in Tokyo still send printed invitations to all eligible recipients. Moreover, the checklist developed in this study continues to be used in practice, having been included in a manual issued by the National Federation of Health Insurance Societies and downloaded over 3,000 times from our university website as of February 2025.

A remaining challenge is that the difficulty of making improvements varies depending on the checklist item. Creating appropriate materials involves many elements, and it is not possible to improve them simultaneously. This study demonstrated that four items were relatively easier to improve: “Is there information necessary for the action for health checkups?” “Is there a title and caption to indicate the content of graphics?” “Is the layout consistent and readable?” and “Are there sufficient margins and line spacing?” Among the improved items, three (“Is there information necessary for the action for health checkups?” “Is the layout consistent and readable?,” and “Are there sufficient margins and line spacing?”) were especially emphasized at the beginning of the group session as common pitfalls in existing documents. The other one (“Is there a title and caption to indicate the content of graphics?”) were addressed through specific examples during the category-based explanation of the checklist—for instance, suggesting that captions be added to unfamiliar local mascots to avoid confusion. These items were emphasized during the group session, which likely contributed to the improvements. When developing future training programs, focusing on these easier-to-improve items in the initial training could facilitate more effective material creation. However, there were four items that were rated as ‘not suitable,’ both before and after the training: “Are the benefits of undergoing health checkups explained?” “Is the text easy to read?” “Are the meanings of the graphic illustrations, lists, tables, or charts clear with favorable emphasis?” and “Is the communication not one-sided?” The training provided in this study may have been insufficient to address these aspects, indicating the need for new strategies and measures such as implementing advanced training sessions that include techniques to emphasize the benefits to the reader, balancing clarity with accuracy, effectively using charts and illustrations, and developing strategies to keep the reader engaged.

This study did not evaluate recipients’ perspectives on the revised materials or examine differences in health checkup rates based on participation in the training. These perspectives are important for assessing the alignment between recipient perceptions and the checklist, and should be addressed in future research.

## 5 Conclusion

Although the suitability scores increased after training, the difference was not statistically significant. However, improvements in specific items suggest a potential benefit of the training.

## Data Availability

The raw data supporting the conclusions of this article will be made available by the authors, without undue reservation.
