# Supplementary material for: Development and evaluation of training programs to improve health checkup recommendation materials
Source: Front Public Health. 2025 Sep 3;13:1540529. doi: 10.3389/fpubh.2025.1540529 (PMC12440992; doi:10.3389/fpubh.2025.1540529)
Supplement: Supplementary file 1 [file Table_1.docx]

Supplementary Material

**Supplementary Table 1.** Evaluators and allocation

| Insurer ID | Pre-training material | | | Post-training material | | |
| --- | --- | --- | --- | --- | --- | --- |
| 1 | A | B | C | D | E | F |
| 2 | A | B | C | D | E | F |
| 3 | F | A | B | C | D | E |
| 4 | C | D | E | F | A | B |
| 5 | E | F | A | B | C | D |
| 6 | B | C | D | E | F | A |
| 7 | D | E | F | A | B | C |
| 8 | A | B | C | D | E | F |
| 9 | C | D | E | F | A | B |
| 10 | F | A | B | C | D | E |
| 11 | B | C | D | E | F | A |
| 12 | E | F | A | B | C | D |
| 13 | D | E | F | A | B | C |
| 14 | A | B | C | D | E | F |
| 15 | C | D | E | F | A | B |
| 16 | F | A | B | C | D | E |
| 17 | B | C | D | E | F | A |
| 18 | E | F | A | B | C | D |
| 19 | D | E | F | A | B | C |
| 20 | A | B | C | D | E | F |
| 21 | C | D | E | F | A | B |
| 22 | F | A | B | C | D | E |
| 23 | B | C | D | E | F | A |
| 24 | E | F | A | B | C | D |
| 25 | D | E | F | A | B | C |
| 26 | A | B | C | D | E | F |
| 27 | C | D | E | F | A | B |
| 28 | F | A | B | C | D | E |
| 29 | B | C | D | E | F | A |
| 30 | E | F | A | B | C | D |
| 31 | D | E | F | A | B | C |
